# Evaluating the efficacy of the WHO QualityRights e-training in promoting the rights of persons with mental health conditions and psychosocial disabilities: a cluster randomised controlled trial in Ghana

**DOI:** 10.1136/bmjgh-2025-021215

**Published:** 2025-12-09

**Authors:** Maria Francesca Moro, Leveana Gyimah, Ezra Susser, Joana Ansong, Jeremy C Kane, Akwasi Osei, Oye Gureje, Humphrey Kofie, Dan Taylor, Natalie Drew, Sally-Ann Ohene, Abdul Fatawu, Nii Lartey Addico, Michela Atzeni, Silvia D’Oca, Michelle Funk, Mauro Giovanni Carta

**Affiliations:** 1 World Health Organization, Geneva, Switzerland; 2 Columbia University Mailman School of Public Health, New York, New York, USA; 3 WHO Country Office for Ghana, Accra, Ghana; 4 New York State Psychiatric Institute, New York, New York, USA; 5 Mental Health Authority, Ghana Ministry of Health, Accra, Ghana; 6 WHO Collaborating Centre for Research and Training in Mental Health, Neurosciences and Substance Abuse, Department of Psychiatry, University of Ibadan, Ibadan, Nigeria; 7 Mental Health Society of Ghana, Accra, Ghana; 8 MindFreedom Ghana, Accra, Ghana; 9 University of Cagliari, Cagliari, Italy

**Keywords:** mental health & psychiatry, health services research, global health, health education and promotion, Africa South of the Sahara

## Abstract

**Introduction:**

People with mental health conditions and psychosocial disabilities frequently experience human rights violations. This study assessed the efficacy of the WHO QualityRights e-training in promoting their rights within the mental healthcare system.

**Methods:**

In this cluster-randomised trial in three psychiatric hospitals in Ghana, we randomly selected units within each hospital and randomised them 1:1 to the intervention (WHO QualityRights e-training) or control arm (COVID-19 e-training). The intervention included presentations, videos, interactive exercises and forum discussions. Mental health professionals in these facilities were eligible participants. Primary outcomes were changes in knowledge about the rights of persons with mental health conditions and in the attitudes towards them, measured post-intervention using the WHO QualityRights Knowledge and Attitudes questionnaires. Secondary outcomes included changes at 3 and 6 months in knowledge, attitudes and mental health professionals’ practices related to substitute decision-making and coercion. Data analysts were masked to group assignment.

**Results:**

Between 11 August 2021 and 13 April 2022, 28 clusters (14 per arm) were randomised and 252 participants enrolled (126 per arm); 179 (71%) were women. 206 (81.8%) completed the post-training follow-up. The intervention group showed significantly greater improvements in scores on the WHO QualityRights Knowledge (mean difference: 4.61 [95% CI 3.49 to 5.72], d=1.12) and Attitudes (−7.99 [95% CI −10.32 to −5.66], d=0.92) compared with the control group, with similarly significant results at 3 and 6 months. Additionally, intervention participants reported less frequent use of substitute decision-making and restraint (−2.60 [95% CI −4.05 to −1.16], d=0.52 at 3 months; −1.76 [95% CI −3.11 to −0.40], d=0.36 at 6 months).

**Conclusion:**

This study showed that the WHO QualityRights e-training effectively improves mental health professionals’ knowledge and attitudes and can lead to reduction in providers’ use of substitute decision-making and coercion practices, thus suggesting a need for improved investment in rights-based interventions and further research.

**Trial registration number:**

NCT04728243.

**WHAT IS ALREADY KNOWN ON THIS TOPIC**

- Most interventions to promote the rights of persons with mental health conditions and psychosocial disabilities in healthcare settings focus on education or stigma reduction, but few align fully with the United Nations Convention on the Rights of Persons with Disabilities (UNCRPD) or assess long-term, rights-based behavioural change.

**WHAT THIS STUDY ADDS**

- This cluster randomised controlled trial is the first to rigorously evaluate the WHO QualityRights e-training—uniquely grounded in the UNCRPD and co-developed with persons with mental health conditions and psychosocial disabilities—using validated measures and long-term follow-up.
- The study showed that the training effectively improves mental health professionals’ knowledge about human rights and attitudes towards persons with mental health conditions and psychosocial disabilities and their role as rights holders, and importantly, provides the first empirical evidence that such training can reduce the use of substitute decision-making and coercion in mental health practice.

**HOW THIS STUDY MIGHT AFFECT RESEARCH, PRACTICE OR POLICY**

- Given the impact human rights violations have on the health of people with mental health conditions and psychosocial disabilities, an effort is needed to carry out methodologically strong research that will provide robust evidence to support further investment in interventions such as the QualityRights e-training and make steps forward promoting the rights of people with mental health conditions and psychosocial disabilities.

## Introduction

 People with mental health conditions and psychosocial disabilities are frequently exposed to human rights violations within the mental healthcare system.^1^,^6^ In most countries, the primary providers of mental healthcare are long-stay psychiatric institutions,^6 7^ where persons can experience poor conditions of the physical infrastructure, inadequate sanitation and overcrowding.^2 7^ In many mental health facilities, people continue to be chained,^25 8^,^12^ verbally abused^413^,^18^ and sedated for control.^719^,^22^ Seclusion and restraints, despite their severe risks to health and safety,^23 24^ remain common,^25^,^28^ often used as a punishment.^29 30^ The prevalence of these violations is high in both high-income and low- and middle-income countries, and thus cannot be explained simply by a lack of resources.^31^ Weak or absent monitoring mechanisms within mental health facilities allow these violations to persist, hidden from the public eye. Beyond ethical concerns, human rights violations have profound repercussions on the health of persons with mental health conditions and psychosocial disabilities. The relationship between human rights violations and mental health is complex and bidirectional: mental health conditions increase vulnerability to human rights violations, and, in a vicious cycle, human rights violations negatively impact mental health. Alarmingly, these violations often occur in psychiatric institutions—the very places meant to provide care and support—highlighting the urgent need for mental health system reform. A key aspect of this reform is changing providers’ practices that may lead to human rights violations, as mental health professionals are the first point of contact with formal services for people with mental health conditions and psychosocial disabilities when they experience moments of distress. However, two main barriers hinder progress. The first barrier is the lack of human rights literacy among health professionals, including mental health professionals.^32 33^ Historically, the health and the human rights fields have operated separately due to differing philosophical perspectives, languages and methods of work.^34 35^ Many health professionals have resisted incorporating a human rights perspective into their work, doubting its applicability and utility.^35^ This is reflected in the current scarcity of training programmes and curricula on human rights in mainstream education for health professionals, leading to limited awareness of service users’ rights and providers’ responsibilities.^33^ The second barrier is the stigma and negative attitudes among mental health professionals towards people with mental health conditions and psychosocial disabilities and their role as rights holders. One might assume that mental health providers, who undergo years of training and have knowledge about mental health issues, would hold positive—or at least neutral—attitudes towards people with mental health conditions. However, many studies suggest that this is not always true.^36^,^40^

Overall, currently, there is consensus among health professionals on the importance of a human rights-based approach in healthcare, as it is seen as fundamental for positive therapeutic outcomes and promoting well-being.^41^ However, in mental health, many providers have reported challenges about fully integrating this approach into daily practice.^42^ In particular, mental health providers and their organisations, with some exceptions, have questioned the feasibility of implementing certain provisions outlined in the United Nations Convention on the Rights of Persons with Disabilities (UNCRPD) and its General Comment on Article 12,^43^ which demand to eliminate substitute decision-making approaches (where decisions are based on what is deemed to be in the ‘best interest’ of individuals and not on their will and preferences) and coercion—on the basis of disability.^44^,^49^ Conversely, international human rights bodies and organisations of people with psychosocial disabilities strongly advocate for these provisions to be implemented.

To address this situation, the World Health Organization (WHO) launched the QualityRights (QR) e-training,^50^ which was developed in partnership with different stakeholders, including psychiatrists and people with lived experience of mental health conditions and psychosocial disabilities. The training applies the framework of the UNCRPD to increase knowledge about the rights of persons with mental health conditions and psychosocial disabilities and change the negative attitudes towards them and their role as rights holders. Furthermore, it provides mental health professionals (among other stakeholders) with the skills necessary to implement a human rights-based approach in mental health. Currently, there is a growing interest in the QR e-training, and WHO has been asked to implement it in multiple countries.^51^ However, the efficacy of this intervention has not been rigorously evaluated.

This cluster randomised controlled trial (cRCT) aims to determine the efficacy of the QR e-training compared with a placebo intervention in improving mental health professionals’ knowledge of human rights and attitudes towards people with psychosocial disabilities and mental health conditions and their role as rights holders. Furthermore, this study evaluates whether the QR e-training is effective in changing substitute decision-making and coercion practices among mental health providers. The trial was conducted in Ghana, a country that had come under scrutiny for human rights violations in mental health facilities.^8 16 52^ A cRCT design was chosen to account for potential differences between hospital units (clusters) and avoid contamination.

## Methods

### Study design

The study was a cRCT with participants recruited from mental health professionals working in three large psychiatric hospitals in Ghana: Accra, Pantang and Ankaful (online supplemental material 1). The hospital units served as the clusters randomised to the intervention and control arms.

In Ghana, mental health services are predominantly available in urban areas, provided either through the three large psychiatric hospitals selected for this study or smaller psychiatric units within three teaching hospitals and five regional hospitals.^53^ These hospitals were included in a nationwide assessment of care quality and respect for human rights conducted in Ghana’s mental health services in 2020.^53 54^

Persons with mental health conditions and psychosocial disabilities and their representative organisations were engaged in the design and conduct of the present study.

### Randomisation and masking

**Figure 1:**
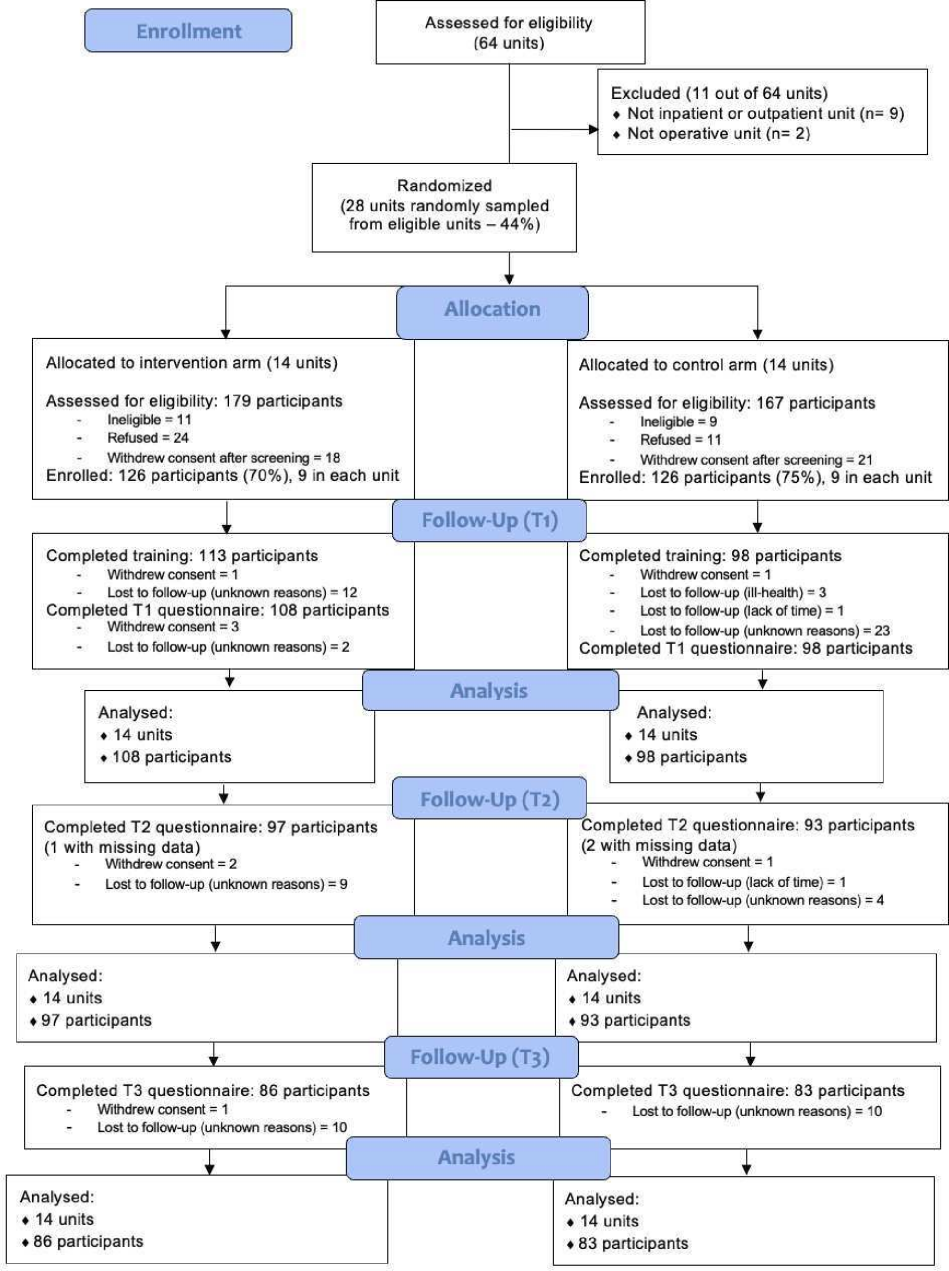
Consolidated Standards of Reporting Trials (CONSORT) flow diagram.

Clusters and participants were selected in four steps. First, the 64 units (28 from Accra, 22 from Pantang and 14 from Ankaful) across the three psychiatric hospitals were screened for eligibility (figure 1). Second, an independent researcher used computer-assisted random sampling to select 28 units (12 from Accra, 10 from Pantang and 6 from Ankaful). Informed consent for cluster participation and randomisation was obtained from the Mental Health Authority (Ministry of Health) and Ghana Health Services in December 2020, and the local trial coordinator managed the enrollment of clusters. Third, within each hospital, the randomly selected units were randomised to the intervention or control arm using a 1:1 allocation ratio. This allocation was carried out by an independent researcher using a computerised randomisation sequence; no restricted randomisation variables were used. Unit randomisation was completed before participant recruitment, and units were not informed of their allocation at the time of participant recruitment. Fourth, the local trial coordinator obtained authorisation from the Mental Health Authority (Ministry of Health) to access the register of mental health professionals employed in the three hospitals. In each randomised unit, a random sample of potentially eligible mental health professionals identified from the registers was invited by trained research assistants from the Mental Health Society of Ghana, via email or telephone, to be screened for eligibility. Persons who provided consent to be screened were then recruited upon confirming eligibility. The trained research assistants were blind to the clusters (and thus participants) allocation status to minimise postrandomisation recruitment bias. Recruitment continued until the target number of participants was reached. Participants provided informed consent via the online data collection platform and gained access to the assigned training only after baseline data collection.

Given the nature of the intervention, participants were not blinded to their intervention arm. However, they were not informed of the study hypotheses and only learnt their assigned intervention arm after completing the pre-intervention assessment. The data analysts had access only to anonymised data after each assessment phase.

### Sample size and power calculation

The sample size calculation was based on these assumptions: 0.05 two-sided alpha level, power of 0.80 and ability to detect a 0.5 unit difference (d) in the total score of the WHO QR Attitudes questionnaire (WHO QR Attitudes) between the intervention and control groups. Hospital units served as the randomisation units, with the 28 units randomised with a 1:1 allocation ratio to the intervention or control arms. Based on data from previous cRCT in Ghana,^55 56^ we assumed a 20% loss to follow-up and intraclass correlation coefficient=0.05. We calculated that 126 participants were required in each arm group (nine in each unit).

### Eligibility criteria

Clusters had to be inpatient or outpatient units within the three psychiatric hospitals selected for the study. Units that were not operative at the time of the study (as some units were closed due to the COVID-19 pandemic) were excluded.

Participants had to be 18 years or older, English-speaking, mental health professionals working within the three psychiatric hospitals. Persons who previously participated in the in-person or online WHO QR training were excluded. Only a small number of professionals working in the three psychiatric hospitals had completed the training, so selection bias was considered negligible.

### Interventions in the study

Participants assigned to the intervention arm were enrolled in the QR e-training, designed to increase knowledge about the rights of people with mental health conditions and psychosocial disabilities and change negative attitudes towards these persons as rights holders. The training equips mental health professionals with the skills necessary to promote human rights in mental health. One of the innovative elements of the QR e-training is the active involvement of people with mental health conditions and psychosocial disabilities and their organisations in all phases of the training’s development and implementation. The QR e-training includes six core modules: (1) human rights; (2) human rights, mental health and disability; (3) legal capacity and the right to recovery; (4) ending coercion, violence and abuse; (5) quality services and community inclusion and (6) mental health, well-being and recovery.^57^ Each module consists of presentations, videos, interactive exercises and forum discussions. Special attention in the discussions was given to issues relevant to the Ghanaian context.

Mental health professionals assigned to the control arm were enrolled in the WHO COVID-19 online training series,^58^ which offers a general introduction to COVID-19 and similar respiratory infections, along with guidance on preventive and response measures for facilities and professionals managing COVID-19 cases. This training is shorter than the QR e-training (12 hours 15 min vs 24 hours) and is designed for health professionals and other stakeholders. To evaluate the integrity of this placebo intervention condition, we collected information at each assessment point on potential enrolment in the online QR training or other interventions.

### Fidelity assessment for the intervention

The local trial coordinator and two researchers from the Cagliari University supervised the QR e-training forum discussions at least once per week to ensure quality control and provide detailed feedback. Model fidelity was evaluated through a checklist investigating adherence to topics and time frames. The local trial coordinator and the researchers met weekly to discuss feedback from the training and ensure fidelity was respected throughout the entire training delivery period.

### Instruments and variables used for data collection 

All data were self-reported and collected through an online platform. The sociodemographic questionnaire included the following information: gender; age in years; educational attainment; profession; main language; years working in mental health services; family member(s) with a psychosocial disability or mental health condition; identification as ‘person with a psychosocial disability or mental health condition’.

The primary outcomes, assessed post-training, were the change in knowledge about the rights of persons with psychosocial disabilities/mental health conditions, measured using the WHO’s QR Knowledge questionnaire (WHO QR Knowledge)^59^ and the change in attitudes towards persons with mental health conditions/psychosocial disabilities and their role as rights holders, measured using the WHO QR Attitudes.^59^ Higher scores at this questionnaire indicate more negative attitudes towards people with mental health conditions/psychosocial disabilities and their role as rights holders. These instruments have demonstrated validity and reliability for use in Ghana.^59^

Secondary outcomes (assessed at 3 and 6 months) were changes in knowledge and attitudes, measured as described for the primary outcomes, and change in substitute decision-making and coercion practices in mental health units, measured using the WHO’s QR Practices questionnaire (WHO QR Practices).^59^ This instrument includes two subscales. The first subscale measures how frequently the respondent used practices such as seclusion or restraints over the last 3 months, with higher scores indicating more frequent use. The second subscale evaluates the respondent’s level of agreement with other mental health professionals in their unit regarding the use of these practices. Higher scores indicate that the other professionals in the unit are more inclined to use substitute decision-making and coercion practices than the respondent. This instrument has demonstrated validity and reliability for use in Ghana.^59^ Additionally, we assessed post-training changes at the Seclusion and Restraint Experience Questionnaire (SREQ) (44 items), which measures emotional and ethical experiences related to the use of seclusion and restraint among nurses.^60^ In this study, an abbreviated version (21 items), adapted for the use among mental health professionals, was used.

To ensure quality control, we developed ad hoc questions to collect information on enrolment in the online WHO QR training and any other potential interventions; potential contacts with mental health professionals from other units and participant satisfaction with the training.

### Contamination minimisation

The risk of contamination was minimised through the cluster design. The intervention and control units, although within the same hospital, were distanced and physically separated. The intervention occurred during the COVID-19 pandemic, a time when hospitals’ senior management prioritised minimising contact between health workers from different units to curb the virus’s spread. This setup made regular contact between participants from the intervention and control clusters negligible. Furthermore, participants were instructed not to share any information about their assigned intervention with mental health professionals in other units. Information on potential contacts with professionals from units allocated to a different arm was collected at the follow-up interviews to assess contamination risk and level.

### Data analytic approach

Descriptive analyses were performed for sociodemographic variables. Univariate analyses examined continuous variables by assessing mean, median and data spread. Tabular analyses were used for categorical variables by assessing frequencies. Missing data were reported for each variable.

Since (1) only the outcome variables had missing values, (2) the proportion of missing data at post-training, 3 months and 6 months follow-up was not large and (3) it seemed plausible the missing-at-random assumption would not introduce substantial bias, we followed Jakobsen *et al* recommendations and decided not to impute for missing data.^61^

An ‘intent-to-treat’ analysis was used to test the study hypotheses. The primary outcomes of interest were (1) changes in knowledge about human rights and (2) changes in the attitudes of mental health professionals towards people with mental health conditions and psychosocial disabilities and their role as rights holders.

Since the data were continuous and longitudinal, linear mixed-effects (LME) regression models were used to estimate average intervention effects while considering the shared variance of repeated measures and the clustered variance. Correlations arising from repeated measures were accounted for by specifying an autoregressive covariance structure. Each outcome was analysed separately using LME. Time, intervention arm and their interaction were included in the model as fixed effects, while participants were evaluated as random effects. Time was entered as a categorical variable with levels corresponding to post-training, 3 months, and 6 months. The effect of the intervention on the outcomes was captured by the interaction between intervention and time, which assessed the mean difference between the intervention arms (QR e-training vs control) in changes of outcomes over time within each group. Full model specifications are provided in online supplemental material 2. Effect sizes were reported as mean differences for each time point and intervention arm, with 95% CIs (and, additionally, as Cohen’s d approximations: d=0.2, 0.5, and 0.8 were considered as small, medium and large effects as per convention).^62 63^

Secondary outcomes included changes in practices and experiences related to substitute decision-making and coercion. Changes in practices were evaluated at 3 and 6 months, while changes in experiences post-intervention. Additionally, changes in knowledge and attitudes were evaluated at 3 and 6 months. These changes were evaluated using the same methodology already described for the primary analyses.

Analyses were performed using SAS V.9.4.

Results reporting followed the Consolidated Standards of Reporting Trials (CONSORT) guidelines.^64^ This trial is registered with ClinicalTrials.gov (NCT04728243).

## Results

Figure 1 shows that 11 out of 64 units were deemed ineligible and excluded from the study. Of the 53 eligible units, 28 (53%) were randomly sampled for enrollment and randomised into the intervention or control arm. In the intervention arm, 179 participants were screened for eligibility: 11 were ineligible, 24 declined participation, 18 withdrew consent after screening, and 126 (70%) were enrolled and completed the pre-intervention (**T_0_**) assessment (table 1). In the control arm, 167 participants were screened: nine were ineligible, 11 declined participation, 21 withdrew consent after screening, and 126 (75%) were enrolled and completed the T_0_ assessment (table 1). Out of the 126 participants, 113 (90%) completed the training in the intervention arm and 98 (78%) in the control arm. Post-intervention follow-up (**T_1_**) was completed by 108 participants (86%) in the intervention arm and 98 (78%) in the control arm. At 3 months, 97 participants (77%) in the intervention arm and 93 (74%) in the control arm completed the follow-up questionnaire. Additionally, at 6 months, 86 participants (68%) in the intervention arm and 83 (66%) in the control arm completed the follow-up questionnaire. Recruitment was completed between 11 August 2021 and 13 April 2022, while the last 6-month follow-up occurred in January 2023.

**Table 1:** Comparison of sociodemographic characteristics at baseline between the intervention and control arm

Intervention (n=126)	Control (n=126)
**Gender**
Woman	89 (70.63)	90 (71.43)
Man	37 (29.37)	36 (28.57)
Other gender not listed	0	0
**Age**
33.57 (5.48)	33.20 (5.10)
**Education**
Less than high school degree	3 (2.38)	0
High school degree or equivalent	6 (4.76)	10 (7.94)
Vocational/Technical school (2 years)	0	3 (2.38)
Some college	30 (23.81)	21 (16.67)
College graduate (4 years)	44 (34.92)	57 (45.24)
Master’s degree	5 (3.97)	4 (3.17)
Doctoral degree (PhD)	0	0
Professional degree (MD, JD, etc)	9 (7.14)	9 (7.14)
Other	29 (23.02)	22 (17.46)
**Profession**
Medical doctor	5 (3.97)	4 (3.17)
Nurse	104 (82.54)	117 (92.86)
Health assistant	2 (1.59)	1 (0.79)
Physician assistant	4 (3.17)	1 (0.79)
Midwife	10 (7.94)	1 (0.79)
Other	1 (0.79)	2 (1.59)
**Main language**
Akan (Fante, Asante Twi and Akuapem Twi)	46 (36.51)	45 (35.71)
Akposo	0	0
Dagaare	1 (0.79)	0
Dagbani	1 (0.79)	1 (0.79)
Dangme	3 (2.38)	3 (2.38)
English	59 (46.83)	61 (48.41)
Ewe	7 (5.56)	8 (6.35)
Ga	5 (3.97)	6 (4.76)
Gonja	0	0
Kasem	0	0
Nzema	0	1 (0.79)
Other	4 (3.17)	1 (0.79)
**Years working in a mental health service**
7.09 (5.10)	7.37 (4.77)
**Person with a psychosocial disability or mental health condition in the close family**
Yes	25 (19.84)	14 (11.11)
No	101 (80.16)	112 (88.89)
**Participant identifies as a person with a psychosocial disability or mental health condition**
Yes	21 (16.67)	22 (17.46)
No	105 (21)	104 (82.54)
**WHO QR Knowledge—total score**
10.43 (3.97)	10.87 (4.25)
**WHO QR Attitudes—total score**
47.45 (9.29)	47.72 (9.32)
**WHO QR Practices**
Subscale 1	12.31 (5.58)	10.25 (4.96)
Subscale 2	12.74 (3.09)	12.91 (3.37)
**SREQ**
Negative experiences	11.63 (3.37)	11.75 (3.67)
Positive experiences	5.33 (1.76)	5.35 (1.70)
Experiences of control	9.23 (2.38)	9.34 (2.25)
Experiences of duty	6.33 (1.56)	6.25 (1.48)
Experiences of misuse of S/R	10.61 (2.89)	10.13 (2.92)
Experiences of S/R as good practice	10.79 (2.53)	10.99 (2.46)

**S/R**, seclusion and restraint; **SREQ**, Seclusion and Restraint Experience Questionnaire; **WHO QR Attitudes**, WHO QualityRights Attitudes Questionnaire; **WHO QR Knowledge**, WHO QualityRights Knowledge Questionnaire; **WHO QR Practices**, WHO QualityRights Practices Questionnaire.

Table 2 shows that participants in the intervention arm had greater improvements in WHO QR Knowledge total mean scores post-training (primary outcome) than participants in the control arm (mean difference: 4.61 [95% CI 3.49 to 5.72]). This finding indicates that participants who completed the WHO QR e-training had increased knowledge of human rights compared with participants who completed the WHO COVID-19 training (post-intervention). Similarly, participants in the intervention arm showed greater improvements in WHO QR Attitudes total mean scores post-training (primary outcome) than participants in the control arm (mean difference: −7.99 [95% CI −10.32 to −5.66]). This finding indicates that, after completing the WHO QualityRights e-training, participants in the intervention arm had less negative attitudes towards persons with mental health conditions and psychosocial disabilities compared with the control group (post-intervention).

**Table 2:** Outcomes post-training (T_1_), at 3 months (T_2_), and at 6 months (T_3_)—mean differences between the two arms (compared with baseline) for each time point

	Intervention arm Mean	Control arm Mean	Mean difference (95% CI)	Cohen’s d
**WHO QR Knowledge**				
Baseline (T_0_)	10.43	10.87		
Post-training (T_1_)	15.26	11.07	4.61 (3.49 to 5.72)	1.12
At 3 months (T_2_)	13.50	10.96	2.97 (2.03 to 3.92)	0.72
At 6 months (T_3_)	12.87	10.83	2.47 (1.44 to 3.51)	0.60
**WHO QR Attitudes**				
Baseline (T_0_)	47.45	47.72		
Post-training (T_1_)	37.69	45.99	−7.99 (−10.32 to −5.66)	0.92
At 3 months (T_2_)	39.91	46.54	−6.36 (−8.72 to −4.00)	0.78
At 6 months (T_3_)	40.44	47.15	−6.43 (−8.81 to −4.05)	0.74
**WHO QR Practices—subscale 1**				
Baseline (T_0_)	12.31	10.25		
Post-training (T_1_)	NA	NA	NA	NA
At 3 months (T_2_)	9.85	10.39	−2.60 (−4.05 to −1.16)	0.52
At 6 months (T_3_)	10.51	10.20	−1.76 (−3.11 to −0.40)	0.36
**WHO QR Practices—subscale 2**				
Baseline (T_0_)	12.74	12.91		
Post-training (T_1_)	NA	NA	NA	NA
At 3 months (T_2_)	13.38	12.39	1.17 (0.66 to 2.27)	0.35
At 6 months (T_3_)	13.32	12.93	0.56 (−0.57 to 1.70)	0.15
**SREQ—negative experiences**				
Baseline (T_0_)	11.63	11.75		
Post-training (T_1_)	13.43	11.28	2.26 (1.30 to 3.23)	0.68
At 3 months (T_2_)	NA	NA	NA	NA
At 6 months (T_3_)	NA	NA	NA	NA
**SREQ—positive experiences**				
Baseline (T_0_)	5.33	5.35		
Post-training (T_1_)	4.17	4.86	−0.67 (−1.16 to −0.18)	0.41
At 3 months (T_2_)	NA	NA	NA	NA
At 6 months (T_3_)	NA	NA	NA	NA
**SREQ—experiences of control**				
Baseline (T_0_)	9.23	9.34		
Post-training (T_1_)	6.93	8.38	−1.34 (−2.08 to −0.59)	0.59
At 3 months (T_2_)	NA	NA	NA	NA
At 6 months (T_3_)	NA	NA	NA	NA
**SREQ—experiences of duty**				
Baseline (T_0_)	6.33	6.25		
Post-training (T_1_)	4.95	5.68	−0.81 (−1.31 to −0.31)	0.53
At 3 months (T_2_)	NA	NA	NA	NA
At 6 months (T_3_)	NA	NA	NA	NA
**SREQ—experiences of misuse of S/R**				
Baseline (T_0_)	10.61	10.13		
Post-training (T_1_)	11.03	9.42	1.13 (0.25 to 2.01)	0.40
At 3 months (T_2_)	NA	NA	NA	NA
At 6 months (T_3_)	NA	NA	NA	NA
**SREQ—experiences of S/R as good practice**				
Baseline (T_0_)	10.79	10.99		
Post-training (T_1_)	9.34	10.33	−0.80 (−1.51 to −0.08)	0.28
At 3 months (T_2_)	NA	NA	NA	NA
At 6 months (T_3_)	NA	NA	NA	NA

The observed numbers are shown in figure 1.

**NA**, not applicable; **S/R**, seclusion and restraint; **SREQ**, Seclusion and Restraint Experience Questionnaire; **WHO QR Attitudes**, WHO QualityRights Attitudes Questionnaire; **WHO QR Knowledge**, WHO QualityRights Knowledge Questionnaire; **WHO QR Practices**, WHO QualityRights Practices Questionnaire.

A strong difference in scores post-training was also observed between the intervention and control arm in the SREQ subscales (table 2). Compared with participants in the control arm, participants in the intervention arm reported more negative experiences (mean difference: 2.66 [95% CI 1.30 to 3.23]) and less positive experiences (mean difference: −0.67 [95% CI −1.16 to −0.18]) related to using seclusion and restraints. Similarly, compared with participants in the control arm, participants in the intervention arm were less likely to view seclusion and restraint as acceptable means to manage unsettled situations or service users’ behaviours (mean difference: −1.34 [95% CI −2.08 to −0.59]), less likely to consider seclusion and restraint essential components of their work and duty (mean difference: −0.81 [95% CI −1.31 to −0.31]), more likely to report misuse of seclusion and restraint within their units (mean difference: 1.51 [95% CI 0.54 to 2.48]) and less likely to regard seclusion and restraint as good mental health practices (mean difference: 1.51 [95% CI 0.54 to 2.48]).

Seventy-four (68.52%) participants in the intervention arm and 62 (63.27%) in the control arm reported being very satisfied with the training, 31 (28.70%) and 34 (34.69) being satisfied, while three (2.78%) and two (2.04%) being neither satisfied nor dissatisfied.

At both 3 months (T_2_) and 6 months (T_3_), participants in the intervention arm continued to show increased knowledge of human rights and more positive attitudes towards persons with psychosocial disabilities and mental health conditions compared with participants in the control arm, with mean differences of 2.97 [95% CI 2.03 to 3.92] and −6.36 [95% CI −8.72 to −4.00] at 3 months, and 2.47 [95% CI 1.44 to 3.51] and −6.43 [95% CI −8.81 to −4.05] at 6 months, respectively (table 2). Furthermore, participants in the intervention arm reported use of substitute decision-making and restraint practices less frequently than participants in the control arm, with a mean difference of −2.60 [95% CI −4.05 to −1.16] at 3 months and −1.76 [95% CI −3.11 to −0.40] at 6 months. At 3 months, participants in the intervention arm perceived their colleagues as more willing to use substitute decision-making and coercion than themselves (mean difference: 1.17 [95% CI 0.66 to 2.27]), but by 6 months there were no differences between the two groups (mean difference: 0.56 [95% CI −0.57 to 1.70]).

Overall, the information collected on potential contacts with mental health professionals in units allocated to a different arm (at T_1_, T_2_, T_3_) suggests a low level of contamination.

## Discussion

To our knowledge, this is the first study to evaluate the efficacy of a training not only in improving mental health professionals’ knowledge about human rights and attitudes towards persons with mental health conditions and psychosocial disabilities (and their role as rights holders), but also in changing their practices related to substitute decision-making and coercion. While many studies have explored the effect of educational interventions on mental health professionals’ knowledge and attitudes,^65^ existing interventions often do not align with the human rights framework of the UNCRPD. These programmes were predominantly developed by health practitioners, often reflecting their perspective, even when this contradicted the UNCRPD principles and the views of people with mental health conditions and psychosocial disabilities. In our view, education on human rights and interventions to address negative attitudes should be based on the UNCRPD and reflect the perspectives of people with mental health conditions and psychosocial disabilities and their representative organisations. This is the approach adopted by the WHO QR e-training evaluated in this study. Another limitation of past initiatives to address stigma is their primary focus on changing mental health professionals’ negative attitudes towards people with mental health conditions in general.^65^ In contrast, the WHO QR e-training focuses on changing also attitudes towards people with psychosocial disabilities’ role as rights holders, a key aspect for promoting their full participation in society. Among all the studies to address health professionals’ stigma, only two^66 67^ assessed behavioural changes. However, neither assessed changes in behaviours related to substitute decision-making and coercion, as was done in this study.

This trial demonstrated that the QR e-training, compared with a placebo intervention, is strongly effective in improving mental health professionals’ knowledge about human rights and attitudes towards persons with mental health conditions and psychosocial disabilities and their role as rights holders. Furthermore, these improvements appear to be maintained longer-term (at 3 and 6 months). Additionally, the QR e-training effectively reduced self-reported practices related to substitute decision-making and restraint at 3 and 6 months, although this effect seems to decrease over time. This may be attributable to the intervention of senior management at the psychiatric hospitals: after completing the QR e-training, some nurses reportedly began refusing to use seclusion and restraints in their units. In response, senior management held meetings to challenge this shift and nurses were strongly advised to follow the Ghana Mental Health Act provisions on the use of seclusion and restraint, which permit these practices in a variety of situations. This highlights the importance of involving senior management and unit heads in QR training before extending it to all staff in a psychiatric facility, to facilitate working towards eliminating these practices in mental health services. Interestingly, participants who completed the QR e-training were more likely to perceive their colleagues as more inclined to use substitute decision-making and coercion than themselves at 3 but not at 6 months. This may be due to senior management’s intervention or, as we hope, it may suggest that participants who completed the QR e-training shared their knowledge and skills with colleagues, reducing their use of substitute decision-making and coercion, thereby amplifying the training’s effect. While our findings provide strong evidence of the WHO QR e-training’s efficacy in improving mental health professionals’ knowledge, attitudes and practices in Ghana, further research is needed to test the applicability of this approach in other countries and with different target populations (eg, persons with mental health conditions and psychosocial disabilities, family members and caregivers, general health workers) and its sustainability for longer periods of time.

Some limitations should be considered when interpreting the results of this study. First, the data analysed are from a cRCT. Conducting a cRCT always poses challenges like inadequate follow-up. These challenges were amplified in this study, as participants were mental health workers providing services during a pandemic, possibly increasing loss to follow-up. However, since follow-up loss was similar in both groups and unlikely related to the training content or the evaluated outcomes, it is unlikely that this loss introduced bias in the effect estimates. Second, the intervention tested had a limited scope, focusing on increasing mental health providers’ knowledge about the rights of persons with mental health conditions and changing negative attitudes towards them. The training also aimed to equip professionals with skills to improve their practices related to substitute decision-making and coercion. However, these outcomes, particularly substitute decision-making and coercion practices, are influenced by external factors, including workplace practices and local laws and policies. Despite these constraints, the goal was to show that the QR intervention could still meaningfully improve knowledge, attitudes and practices, and thus quality of services in mental health facilities. Third, our study was conducted in a specific setting (three psychiatric hospitals in Ghana) and with a specific target (mental health professionals). It would be important to evaluate the efficacy of the QR e-training in other settings and countries and among different stakeholder groups to understand the generalisability of the findings. This study has also several strengths. First, and most importantly, the whole project was developed and implemented with the active participation of people with mental health conditions and psychosocial disabilities and their organisations. This aligns with the UNCRPD requirement of full inclusion of persons with disabilities in all processes that may affect them and recent calls to shift the focus from research ‘about’ people with disabilities to research ‘with’ people with disabilities. Second, this study was conducted in Ghana in close collaboration with the Ghanaian government and local organisations, boding well for the continuation of this initiative beyond the trial’s completion. Third, this study addressed some limitations of past research. It is an RCT. In contrast to previous studies, which often relied solely on pre-post intervention evaluations, this trial followed participants over 6 months, with multiple assessment points. Furthermore, in this study, breaking with the past, measures tested for reliability and validity were used (including a measure on behavioural changes, an area neglected in prior research).

## Conclusions

This study showed that the WHO QualityRights e-training effectively improves mental health professionals’ knowledge about human rights and attitudes towards persons with mental health conditions and psychosocial disabilities (and their role as rights holders). Importantly, it provides the first empirical evidence that this training can reduce mental health professionals’ use of substitute decision-making and coercion practices.

Given the impact that human rights violations in mental health settings have on persons with psychosocial disabilities and mental health conditions, there is a pressing need for methodologically rigorous research in this area. This study offers robust evidence supporting further investment in interventions like the QR e-training and makes steps forward promoting the rights of persons with psychosocial disabilities and mental health conditions within psychiatric settings.

## Supplementary material

10.1136/bmjgh-2025-021215 online supplemental file 1

10.1136/bmjgh-2025-021215 online supplemental file 2

## Data Availability

Data are available on reasonable request.
