# Supplementary material for: Evaluating the efficacy of the WHO QualityRights e-training in promoting the rights of persons with mental health conditions and psychosocial disabilities: a cluster randomised controlled trial in Ghana
Source: BMJ Glob Health. 2025 Dec 9;10(12):e021215. doi: 10.1136/bmjgh-2025-021215 (PMC12699628; doi:10.1136/bmjgh-2025-021215)
Supplement: online supplemental file 1 [file bmjgh-10-12-s001.docx]

Supplemental material 1. Profile of the three psychiatric hospitals where the study took place

| **Mental health**  **facility** | **N. of**  **Staff members*** | **No. of**  **Service users**** | **Bed capacity** | **Units** |
| --- | --- | --- | --- | --- |
| **Accra Psychiatric**  **Hospital** | 782 | 319 | 600 | 28 |
| **Ankaful Psychiatric Hospital** | 462 | 236 | 350 | 22 |
| **Pantang Psychiatric Hospital** | 703 | 190 | 500 | 14 |

* Data on in-patient and out-patient services provided by the Mental Health Authority (Ministry of Health of Ghana).

**Data on in-patient units’ service users (per day), provided by the Mental Health Authority (Ministry of Health of Ghana).
