# Supplementary material for: Evaluating the efficacy of the WHO QualityRights e-training in promoting the rights of persons with mental health conditions and psychosocial disabilities: a cluster randomised controlled trial in Ghana
Source: BMJ Glob Health. 2025 Dec 9;10(12):e021215. doi: 10.1136/bmjgh-2025-021215 (PMC12699628; doi:10.1136/bmjgh-2025-021215)
Supplement: online supplemental file 2 [file bmjgh-10-12-s002.docx]

Supplemental material 2. General model statement for the Random intercept with two group-level covariates and interaction.

***Level 1:***

$Y_{ij}=b_{0j}+\varepsilon_{ij}$

***Level 2:***

b_0j_ = β_00_ +$\beta\text{01}w\text{j}+ \beta\text{02}t\text{j}+ \beta\text{03}w\text{j}t\text{j}+ b_{0j}^{*}$

***Combined:***

$Y_{ij}=\beta_{00}+ \beta\text{01}wj+ \beta\text{02}t\text{j}+ \beta\text{03}w\text{j}t\text{j} +b_{0j}^{*}+ \varepsilon_{ij}$

- $Y_{\mathrm{ij}}=attitudes$
- $\beta_{00}="average mean" of units mean attitudes$
- $b_{0j}^{*}=difference between \text{specific unit }mean attitu$des and “$average mean” of units mean attitudes$
- $\varepsilon_{\mathrm{ij}}=difference between individual attitudes and unit mean attitudes$
- $t_{j}= time (3=at 6 months;2=at 3 months; 1=post-training;0 baseline)$
- $w_{j}=(1=QR training;0 control)$
- $\beta_{02}=$ regression coefficient for time
- $\beta_{01}=regression coefficient for unit average attitudes when assigned to QR$
- $\beta_{03}=regression coefficient for the interaction of time and intervention$
